# Black widows as plastic wallflowers: female choosiness increases with indicators of high mate availability in a natural population

**DOI:** 10.1038/s41598-020-65985-z

**Published:** 2020-06-02

**Authors:** Catherine E. Scott, Sean McCann, Maydianne C. B. Andrade

**Affiliations:** 1 Department of Biological Sciences, University of Toronto Scarborough, M1C1A4, Toronto, Canada; 2 Present Address: Department of Biology, Acadia University, 33 Westwood Ave. Wolfville, NS, B4P 2R6, Wolfville, Canada

**Keywords:** Behavioural ecology, Sexual selection

## Abstract

Female choice is an important driver of sexual selection, but can be costly, particularly when choosy females risk remaining unmated or experience delays to reproduction. Thus, females should reduce choosiness when mate encounter rates are low. We asked whether choosiness is affected by social context, which may provide reliable information about the local availability of mates. This has been demonstrated in the lab, but rarely under natural conditions. We studied western black widow spiders (*Latrodectus hesperus*) in the field, placing experimental final-instar immature females so they were either ‘isolated’ or ‘clustered’ near naturally occurring conspecifics (≥10 m or ≤1 m, respectively, from a microhabitat occupied by at least one other female). Upon maturity, females in both treatments were visited by similar numbers of males, but clustered females were visited by males earlier and in more rapid succession than isolated females, confirming that proximity to conspecifics reduces the risk of remaining unmated. As predicted, isolated females were less choosy in staged mating trials, neither rejecting males nor engaging in pre-copulatory cannibalism, in contrast to clustered females. These results demonstrate that exposure of females to natural variation in demography in the field can alter choosiness of adults. Thus, female behaviour in response to cues of local population density can affect the intensity of sexual selection on males in the wild.

## Introduction

Female choice is an important driver of sexual selection on males^1,2^. Understanding variation in female choice is valuable because it will influence the strength and direction of sexual selection^3^. Both intrinsic (e.g., age, body condition, mating experience) and extrinsic (e.g., predation risk, density, sex ratio) factors can affect either the feasibility or fitness consequences of exercising choice and cause variation in the expression of mate choice^3–5^. Moreover, since mate choice can impose costs, including time or energy spent sampling, and increased risk of predation or remaining unmated^6–9^, females’ reproductive decisions should depend on the net fitness effects of choice under a given context.

The social environment experienced by animals as juveniles and adults can affect mate choice, including both preferences (rank order in which prospective mates are valued) and choosiness (time or energy individuals are willing to expend on mate assessment)^4^. Attending to sexual signals can provide individuals with information about the local environment, including the availability of potential mates^10^. Exposure to such social information during development can lead to phenotypic plasticity, including behavioural plasticity in the context of mate choice^11–13^. For example, female crickets reared in silence (no cues from males) are more responsive to male signals as adults, and thus less choosy, than females exposed to male signals during development^14^. This type of sexual signal provides social information that reliably indicates the expected availability of potential mates at adulthood^15,16^. In changing environments, conditions experienced as a subadult or in early adulthood may be most relevant to optimal decision-making^17–19^.

One cost of mate choice that is likely to be closely linked with the social environment is the risk of delays to reproduction, or failure to mate at all, when choosy females reject males^20,21^. Females should be less choosy in low-density environments when encounter rates with males are likely to be low^4,22–24^. Demography can vary on relatively small spatial and temporal scales^10,25–28^, and in such situations plasticity in mate choice should be adaptive^29^. For example, an evolutionary history of a risk of mate limitation may lead to unmated females adjusting choosiness as a function of local cues of male availability^30^, and this may include accepting the first available mate regardless of his quality (i.e., the wallflower effect, by which a high risk of remaining unmated decreases female choosiness^21,31^). Most evidence for developmentally-cued mate choice plasticity comes from laboratory experiments^3^. Results from such studies may not always translate to field situations, since in complex social environments females may experience multiple cues simultaneously, and mate availability may fluctuate over small scales. Thus, it is not clear how the social environment during development typically affects the reproductive choice of adult females in nature (but see e.g.^32,33^).

Here we ask how the social environment affects female choice in a field population of western black widow spiders (*Latrodectus hesperus*). In nature, some females mate with multiple males during their reproductive lifetimes^34^ (which can span two mating seasons^35^) and they can exercise choice via mate rejection, including pre-copulatory cannibalism^36^. Males engage in scramble competition^37^, and the pool of sexually active adults is highly male-biased through most of the season^38^. However, there is a substantial risk of females remaining unmated; 4% of wild females were not mated by the end of the 2016 mating season (CES unpublished data). Population density varies across the field site, as spiders may or may not share a microhabitat with conspecifics and the nearest neighbour distance between microhabitats ranges from 0.1–15.6 m (CES unpublished data). Chemical cues produced by nearby males and females^39–41^ are available to *Latrodectus* females throughout their lives and may indicate local population density or proximity of conspecifics^42–44^. However, since adult males abandon their webs and search for females upon maturity^45^ whereas females are sedentary, proximity to conspecific females may provide a more reliable indicator of local population density—and thus expected mate encounter rates—than male cues alone.

We used a field experiment to ask whether female proximity to conspecifics affects mate encounter rates and whether the social environment experienced by females close to maturation affects mate choice. We placed caged subadult females either within 1 m of or at least 10 m from conspecifics in the field (resulting in “clustered” and “isolated” females, respectively). We predicted that females in the clustered treatment would be visited by more males, and would be visited sooner after maturity, compared to isolated females. Thus, we expected that isolated females would perceive both a lower overall density of conspecifics and lower mate availability than clustered females. We predicted that isolated females would be less likely to reject the first male that they encountered in staged mating trials (i.e., be less choosy) than would clustered females. This study investigates female mate choice decisions under exposure to the complex cues typical in nature^46^, allowing us to evaluate plasticity under natural levels and timing of mate availability.

## Results

### Field experiment

Clustered and isolated females attracted a similar total number of males across the 44-day field experiment (Wilcoxon rank sum test: *W* = 214; df = 1; *P* = 0.7; Table 1). Females were rarely visited by males before maturity, and this was equally rare in both treatments (Fisher’s exact test: df = 1; *P* = 0.23; Table 1). There was also no significant difference in the frequency with which clustered and isolated females were visited by at least one male once they were adults (Fisher’s exact test: df = 1; *P* = 0.75). Moreover, a high proportion of females in both treatments had no male visitors as adults (clustered: 35%, n = 17; isolated: 47%, n = 15; Table 1).

**Table 1:** Summary of the number of males arriving at cages of *L. hesperus* females before and after they moulted to maturity, during a 3-month field experiment.

Treatment	n	Before maturity		After maturity	
		Total males	Mean males per female	Total males	Mean males per female
Isolated	15	3	0.2	50	3.3
Clustered	17	0	0	64	3.8
Total	32	3	0.1	114	3.6

Sample sizes include only spiders that matured by the end of the experiment.

**Figure 1:**
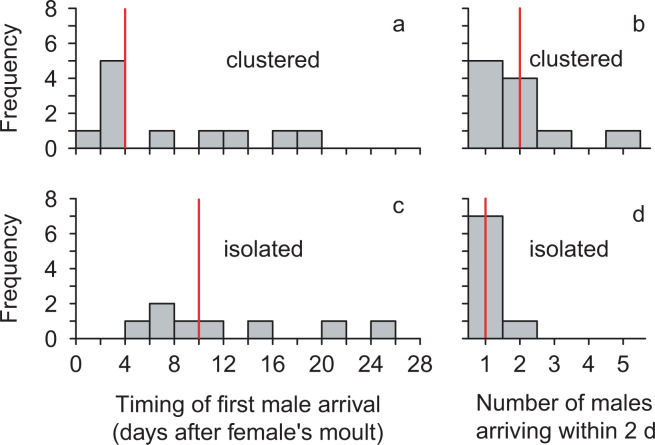
Males arrived earlier and more males arrived in rapid succession at cages of clustered *L. hesperus* females compared to isolated females. Histograms show the day on which the first male (or males) arrived (**a,c**) and the number of males that arrived within two days after the date of the first male’s arrival (**b,d**) at cages of female who were either clustered near conspecific females (**a,b**) or isolated (**c,d**). Vertical red lines = medians.

However, the timing of male arrival at females’ webs following maturation indicates that clustered females could mate sooner and experience a greater opportunity to engage in mate choice compared to isolated females. Males arrived earlier at the webs of clustered adult females (mean [range] = 8.1[1–20] days after maturation; n = 11 females) than at webs of isolated adult females (12.8 [4–26] days after maturation; n = 8 females; *t*-test on log-transformed data: *t* = −1.82, df = 16.9, *P* = 0.043; Fig. 1a,c). Moreover, the number of males that arrived within two days of the first male finding a female was also greater for clustered females (median = 2 males) than for isolated females (median = 1 male; *W* = 63.5; df = 1; *P* = 0.033; Fig. 1b,d).

### Mating trials

**Figure 2:**
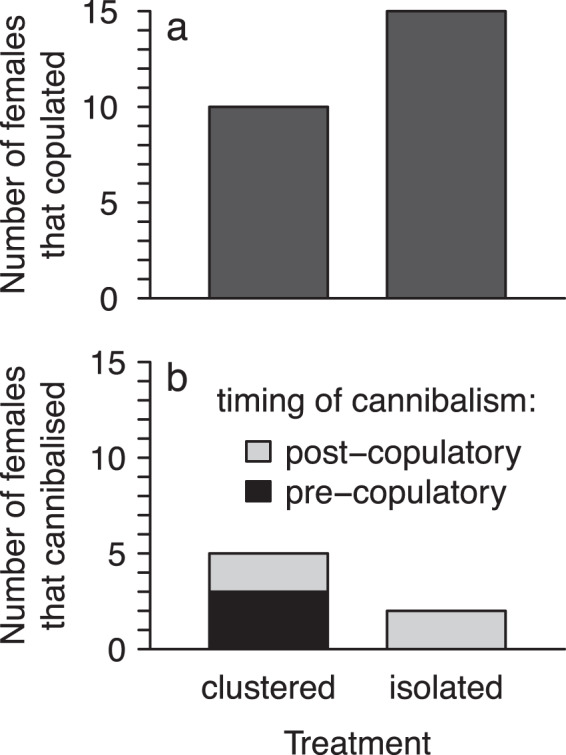
*L. hesperus* females from the clustered treatment rejected males more often than isolated females in staged mating trials. (**a**) Isolated females were more likely than clustered females to copulate at least once (*P* = 0.044). (**b**) Clustered females tended to engage in pre-copulatory cannibalism more often than isolated females (*P* = 0.16).

Females from the clustered treatment were choosier than females from the isolated treatment. When paired with an adult male, clustered females were less likely to copulate and tended to be more cannibalistic than isolated females (Table 2; Fig. 2). Whereas clustered females killed and cannibalized three males during courtship, preventing them from copulating, isolated females never killed courting males. Female age (in days since maturation) did not differ between treatments (*W* = 241, df = 1; *P* = 0.270) and did not affect copulation or cannibalism (Table 2). Moreover, female choosiness (copulation, cannibalism) was not affected by direct experience with male cues after adulthood (measured as the total number of males attracted to female’s cages in the field). The rate of reproductive failure (no offspring produced) for females that copulated at least once did not differ between treatments; 2/15 for isolated females vs. 4/10 for clustered females (Fisher’s exact test; df = 1; *P* = 0.175).

**Table 2:** Results of two logistic regression models (using Firth’s bias reduction method; brglm package in R^65^) assessing whether treatment (clustered or isolated), female age (in days since the moult to maturity), and the number of males who arrived at their web over the course of the field experiment affected two measures of *L. hesperus* female choosiness (copulation and pre-copulatory cannibalism) in mating trials.

Response variable	Predictor variable	Estimate	SE	*Z*	*P*
**Copulation (yes/no)**					
	density treatment	3.49	1.79	2.01	**0.044**
	days since moult	0.08	0.07	1.10	0.272
	number of males	0.09	0.13	0.71	0.481
**Pre-copulatory cannibalism (yes/no)**					
	density treatment	−2.05	1.44	−1.42	0.155
	days since moult	−0.03	0.07	−0.42	0.674
	number of males	−0.002	0.11	−0.02	0.987

## Discussion

These results demonstrate that the social environment experienced by *L. hesperus* females affects their mate choice decisions. Contrary to our predictions, the number of males who visited isolated and clustered females did not differ. Nevertheless, clustered females were visited on average five days earlier and were more likely to be visited by more than one male in rapid succession, which would afford them lowered risk of remaining unmated and greater opportunity to exercise choice compared to isolated females. Neither the number of males who visited females during the experiment nor female age (time spent as an unmated adult) affected female choice. Nonetheless, females from the clustered treatment rejected males more often than isolated females, suggesting that cues of general conspecific density, or proximity, drive variation in choosiness. While similar effects have been shown in other systems, this has rarely been demonstrated in a field population, with the complex cues and social contexts typical of nature^3,46^. These results show that female behaviour in response to cues of local population density can affect the intensity of sexual selection on males in the wild.

Our data agree with predictions that females should decrease choosiness in environments where there is a risk of delayed mating or of failure to mate at all^4,20,21^. Although there was no overall treatment effect on the total number of males that arrived at females’ webs, these data were summed over the entire 44-day observation period. More critical for the female’s behaviour is the time-course of male arrivals, which for isolated females was delayed by five days relative to clustered females’ webs. Such a delay may pose a significant risk of remaining unmated, particularly at the end of the mating season. At this time, the ratio of sexually active males to females (operational sex ratio^47^) shifts rapidly from highly male-biased to slightly female biased^38^, and thus encounter rates with males will decrease. Although females can overwinter and mate or re-mate the following spring^35^, the risk of dying before the next mating season may be greater for unmated females because they suffer decreased longevity relative to mated females^48^. For a female in an environment like that of our isolated treatment, mating indiscriminately with the first male she encounters is likely to be adaptive^4,22^. This will provide fertility assurance but still leaves females with the option of ‘trading-up’ if another, preferred male arrives later^49–51^. Moreover, female widow spiders may be able to bias paternity toward later-mating males via cryptic female choice^52^. Conversely, females in close proximity to conspecifics (as in our clustered treatment) can expect to be visited by multiple males soon after maturity and face a low risk of remaining unmated. For these females, the benefits of exercising choice by rejecting non-preferred males may outweigh the costs^6^.

Sexual cannibalism may be a mechanism of exercising female choice^53^. Only females from the clustered treatment ever engaged in pre-copulatory cannibalism. Although we did not detect a statistically significant difference in cannibalism rates between our treatments, our results appear similar to those of Johnson^54^, who found that female fishing spiders exposed to cues of high male availability during development were more likely to engage in pre-copulatory cannibalism than females housed alone. Sexual cannibalism in *L. hesperus* is rarely observed in mating trials and typically manifests as pre-copulatory cannibalism when females are hungry^36^. This rarity may be exacerbated by the standard method of rearing spiders in isolation from conspecific cues in the laboratory (e.g.^55^).

The timing of male arrival at experimental cages suggests that subadult females do not attract males via volatile cues. Once females matured, however, males found them rapidly (as early as one day after maturity), suggesting that females begin producing sex pheromone immediately upon maturity. Males arrived earlier at female webs in the clustered treatment, possibly because they were near other signaling females (and thus produced a larger combined signal that allowed males to rapidly locate them), or because these females started signaling earlier (in response to signals of nearby conspecific females^56,57^).

We did not measure male phenotypes in this study and so the shape of the females’ preference function is unclear for *L. hesperus*. Previous field experiments at our study site showed that larger males are more likely to find females, but smaller males travel faster during mate searching^38^. This suggests that relatively small males are likely to arrive first at a given female’s web in nature, and larger males later. If isolated females mate indiscriminately with the first male they encounter, this may help to maintain extreme variation in male size in this species^52,58^.

Our experimental design does not allow us to isolate whether the effects demonstrated are due to subadult vs. adult experience, or exposure to male vs. female cues. Further work is needed to determine the critical period for detecting and responding to social cues^16,17,58^. Similarly, it will be particularly valuable to experimentally determine whether the differences in choosiness that we observed were in response to perceived local conspecific density *per se*^59,60^ or simply proximity to signaling females resulting in more rapid male arrival. Mechanisms notwithstanding, these results demonstrate plasticity in female mate choice decisions are shaped by indicators of the availability of mates under complex conditions in the field. Our study allowed us to evaluate plasticity in females exposed to a natural set of cues, and with natural levels and timing of mate availability over a six-week period. Whereas laboratory experiments allow precise analyses of mechanisms underlying this type of plasticity, a field study such as this one provides valuable evidence that such mechanisms manifest in nature, and that they may affect selection in the field.

## Methods

### Natural history and field site

**Figure 3:**
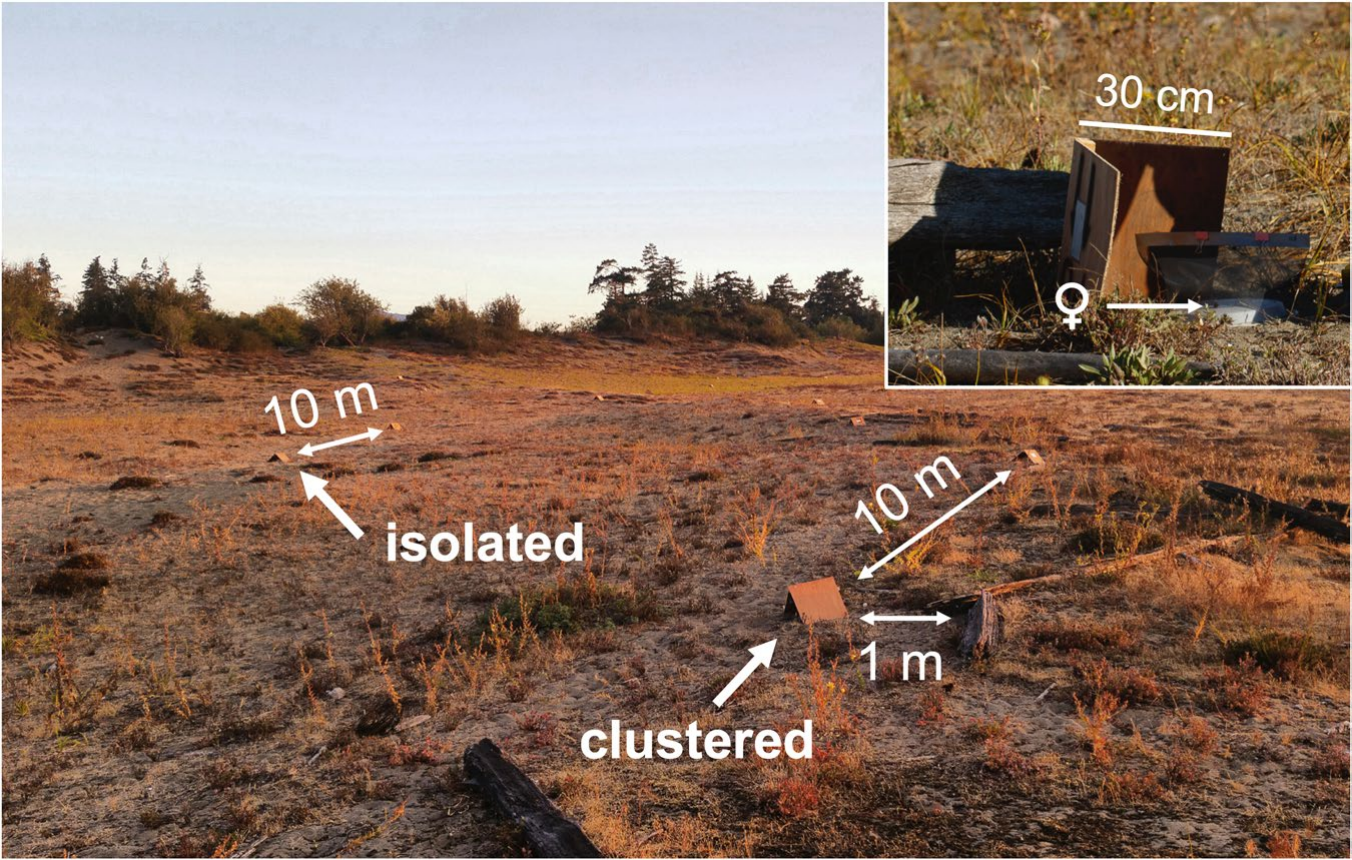
Photograph of a portion of our field site showing the configuration of a study examining the effect of proximity to conspecific females on experimental females during subadulthood and early adulthood. At this site, driftwood logs (seen in centre foreground and at right of main image) provide microhabitats for black widows. Pairs of females were placed at 10-m intervals along a 190-m transect. One female of each pair was isolated (at least 10 m from any naturally occurring females) and the other clustered (within 1 m of a microhabitat containing at least one other female). Each experimental female was housed in a screen cage and placed under an artificial microhabitat (plywood shed) designed to mimic a log (inset, with shed upturned).

We studied a population of *Latrodectus hesperus* Chamberlin and Ivie, 1935 inhabiting the coastal sand dunes above the high-tide line at Island View Beach and T̸IX̱EṈ (Cordova Spit) on the Saanich Peninsula of Vancouver Island, British Columbia, Canada (48.586890, −123.371138). Driftwood logs, other woody debris, and occasional rocks provide microhabitats for western black widows at this site (Fig. 3). The spiders typically spin silk retreats in sheltered areas under logs, and their three-dimensional capture webs extend onto the sand and nearby vegetation. The population is dense, with 2–3 subadult or adult females per square metre of microhabitat in late summer, such that many microhabitats are shared by multiple conspecific females and juveniles (see^35^ for more details about this site and population). However, there is also considerable variability in spatial distribution (nearest neighbour distances between microhabitats range from 0.1–15.6 m), and solitary females are common throughout the season (38.4% [13.8–66.7%] of microhabitats are occupied by a solitary female on any given day across the mating and oviposition season) (CES unpublished data).

Unmated female western black widows produce silk-bound sex pheromones that attract males at long range^38,40,61^ and elicit male courtship behaviour upon contact^62,64^. It is not clear how soon after maturity females begin producing sex pheromone. There is some behavioural evidence for male sex pheromones or chemical cues associated with silk draglines^38,41,62^, and work in a congener indicates that males produce volatile chemical cues that are detectable by other males^42^.

Delays to mating are likely to be costly for *Latrodectus* females. There is evidence for a longevity cost of remaining unmated in a congener (*L. hasselti*^48^). Moreover, since the availability of adult males drops off near the end of the season^38^, females unmated at that point would typically have to survive overwintering and attract a male in the subsequent season in order to reproduce^35^.

### Field experiment

We placed 40 focal females (collected from our field site but outside of the experimental study area) either close to (‘clustered’ treatment) or far from (‘isolated’ treatment) natural microhabitats and monitored them over 44 days during the most active part of the mating season (mid-August to late September). We distributed 20 pairs of cages (‘pheromone traps’ as in^61^) containing immature females (who appeared to be in the penultimate instar based on inspection of their genitalia^45^) and their webs at 10-m intervals along a 190-m transect. Each trap consisted of a mesh-sided cage (design modified from^38^) underneath a wooden shed (two 30 × 30 cm pieces of plywood connected at right angles) that mimicked the natural driftwood microhabitats occupied by black widows at our field site (Fig. 3; similar artificial microhabitats are rapidly colonized by black widows at this site^35^). Both cages and sheds were staked into the ground with bamboo skewers. Each pair of traps included one in an isolated location at least 10 m from the nearest occupied microhabitat (i.e., a log under which there was at least one adult or subadult conspecific female) and one clustered within 1 m of an occupied microhabitat (such that clustered females would have at least one and typically more than one conspecific within a 10 m radius). We chose 10 m for distance between isolated spiders because >90% of microhabitats at our field site are within 10 m of their nearest neighbour (CES unpublished data), so this spacing reflects the natural spatial distribution of microhabitats.

We checked traps each morning and collected all males that had arrived the previous night (males tend to remain on a pheromone trap after they arrive and generally remain on females’ webs until they have the opportunity to copulate; CES pers. obs.). We also checked daily for evidence that experimental females had moulted or mated. When a moult was present in the cage, we noted the date and determined whether the female had moulted to maturity (based on morphology of the external genitalia^45^). After each female had moulted once we determined that eight females were still in the penultimate instar (with closed genitalia), indicating that they had begun the experiment in the antepenultimate instar (isolated: 6 females; clustered: 2 females). If spiders did not reach maturity before the end of the experiment (whether they began in the antepenultimate or penultimate instar) they were excluded from our analysis (isolated: 5 females; clustered: 3 females). We also checked for immature mating^65^ because even though males could not enter females’ cages, the mesh was large enough that it could be possible for a male to copulate with a female through the holes. One female in the isolated treatment apparently mated as an immature (she was observed with open genitalia and then subsequently moulted to maturity). Though we did not ever observe a male outside of her cage, we counted this as a male arrival before maturity (and because we checked immature female genitalia regularly, we are confident that we did not miss any other male visits). We randomly assigned females to treatments and fed all females one house cricket (*Acheta domesticus*) weekly during the experiment to control for effects of female size or condition.

### Mating trials

Following the field experiment, we brought all of the spiders indoors for mating trials. Females remained in the same cages they had inhabited throughout the experiment. We introduced a single male into each of 30 cages (containing 15 adult females from each density treatment). The males were randomly selected from those collected at experimental cages near the end of the experiment. Trials began around 20:00 and proceeded with the lights on for convenience of observation (black widows are nocturnal but they court and copulate normally under white light^63^). We checked each cage approximately every 10 min for copulation or cannibalism until 24:00 (copulation typically lasts longer than 10 min and the first copulation typically occurs within 2 h so we were unlikely to miss matings^65^). We allowed all males to remain in females’ cages all night, terminating trials at 09:00 the following morning. No males that we observed copulating before 24:00 were subsequently cannibalised; post-copulatory cannibalism in this species typically occurs during or immediately following sperm transfer (CES pers. obs). For females that we did not observe copulating, we determined copulation success *a posteriori* by dissecting their genitalia and checking for the presence of sperm plugs, which can only be deposited during copulation (see^52^). If we did not observe copulation prior to noting a cannibalized male, we confirmed that cannibalism was post-copulatory based on the presence of at least one sperm plug and concluded that it was pre-copulatory if there were no plugs present. After the mating trials, all females were brought back to the laboratory in Scarborough and fed one cricket per week until the end of their natural lives. We recorded whether each female produced egg sacs and whether offspring emerged from them to compare the rate of reproductive failure (no fertile eggs produced despite at least one copulation) between treatments.

### Statistical analyses

We analyzed the data using R version 3.6.0^66^. We used a Wilcoxon rank sum test to compare the total number of males attracted by clustered and isolated females during the field experiment, since these data were non-normally distributed and this could not be addressed using a transformation. We used Fisher’s exact tests to ask whether clustered or isolated females were more likely to ever be visited by a male either before or after maturity, because for both of these comparisons there were cells in the contingency tables that were 0 or <5. To assess timing of male arrival at females’ webs, we first log-transformed the number of days until the first male’s arrival (for the subset of females that were ever visited by males) because these data were non-normally distributed and then used a *t*-test to compare the means for each treatment. We also used a Wilcoxon rank sum test to compare the number of males that arrived within two days of the first male’s arrival at each female’s web. This analysis was included as a post-hoc test of the hypothesis that the opportunity for females to choose among males arriving in rapid succession differed between treatments. Males arriving on the same night or over two consecutive nights would provide a female with the opportunity to engage in simultaneous or sequential choice over a short period of time that would cause minimal delays to oviposition.

We used logistic regression with Firth’s bias reduction method (for small samples^67^) to compare copulation and pre-copulatory cannibalism between treatments. We included treatment (isolated or clustered), female age (in days since the moult to maturity), and the number of males who visited each female’s cage during the experiment as predictors in these models. We confirmed that neither female age nor rates of reproductive failure for females who copulated at least once differed between treatments using a Wilcoxon test and Fisher’s exact test, respectively.

## Supplementary information

Supplementary information 1.

Supplementary information 2.

Supplementary information 3.

Supplementary Material.

## Data Availability

All data generated or analysed during this study are included in this article (and its Supplementary Information files).

## References

[1] Darwin, C. The descent of man, and selection in relation to sex (Murray, 1871).

[2] Andersson, M. B. *Sexual selection* (Princeton University Press, 1994).

[3] Ah‐King M, Gowaty PA (2016). A conceptual review of mate choice: stochastic demography, within‐sex phenotypic plasticity, and individual flexibility. Ecol. Evol..

[4] Jennions MD, Petrie M (1997). Variation in mate choice and mating preferences: a review of causes and consequences. Biol. Rev..

[5] Kelly CD (2018). The causes and evolutionary consequences of variation in female mate choice in insects: the effects of individual state, genotypes and environments. Current Opinion in Insect Sci..

[6] Reynolds JD, Gross MR (1990). Costs and benefits of female mate choice: is there a lek paradox?. Am. Nat..

[7] Milinski M, Bakker TC (1992). Costs influences sequential mate choice in sticklebacks, *Gasterosteus aculeatus*. Proc. Roy. Soc. B.

[8] Rowe L (1994). The costs of mating and mate choice in water striders. Anim. Behav..

[9] Booksmythe I, Detto T, Backwell PR (2008). Female fiddler crabs settle for less: the travel costs of mate choice. Anim. Behav..

[10] Kasumovic MM, Brooks RC (2011). It’s all who you know: the evolution of socially cued anticipatory plasticity as a mating strategy. Quart. Rev. Biol..

[11] Pigliucci, M. *Phenotypic plasticity: beyond nature and nurture* (John Hopkins University Press, 2001).

[12] West-Eberhard, M. J. *Developmental plasticity and evolution*. (Oxford University Press, 2003).

[13] Kasumovic MM, Hall MD, Brooks RC (2003). 2012 The juvenile social environment introduces variation in the choice and expression of sexually selected traits. Ecol. Evol..

[14] Bailey NW, Zuk M (2008). Acoustic experience shapes female mate choice in field crickets. Proc. Roy. Soc. B.

[15] Lively CM (1986). Canalization versus developmental conversion in a spatially variable environment. Am. Nat..

[16] Fawcett TW, Frankenhuis WE (2015). Adaptive explanations for sensitive windows in development. Front. Zool..

[17] Sachser N, Kaiser S, Hennessy MB (2013). Behavioural profiles are shaped by social experience: when, how and why. Phil. Trans. Roy. Soc. B.

[18] Cory AL, Schneider JM (2018). Effects of social information on life history and mating tactics of males in the orb‐web spider *Argiope bruennichi*. Ecol. Evol..

[19] Cory AL, Schneider JM (2018). Mate availability does not influence mating strategies in males of the sexually cannibalistic spider *Argiope bruennichi*. PeerJ.

[20] De Jong MC, Sabelis MW (1991). Limits to runaway sexual selection: the wallflower paradox. J. Evol. Biol..

[21] Kokko H, Mappes J (2005). Sexual selection when fertilization is not guaranteed. Evolution.

[22] Bleu J, Bessa-Gomes C, Laloi D (2012). Evolution of female choosiness and mating frequency: effects of mating cost, density and sex ratio. Anim. Behav..

[23] Roff DA, Fairbairn DJ (2015). Bias in the heritability of preference and its potential impact on the evolution of mate choice. Heredity.

[24] Simmons LW, Kvarnemo C (2005). Costs of breeding and their effects on the direction of sexual selection. Proc. Roy. Soc. B.

[25] Gwynne DT, Bailey WJ, Annells A (1998). The sex in short supply for matings varies over small spatial scales in a katydid (*Kawanaphila nartee*, Orthoptera: Tettigoniidae). Behav. Ecol. Sociobiol..

[26] Kasumovic MM, Bruce MJ, Andrade MCB, Herberstein ME (2008). Spatial and temporal demographic variation drives within‐season fluctuations in sexual selection. Evolution.

[27] Punzalan D, Rodd FH, Rowe L (2010). Temporally variable multivariate sexual selection on sexually dimorphic traits in a wild insect population. Am. Nat..

[28] Elias DO, Andrade MCB, Kasumovic MM (2011). Dynamic population structure and the evolution of spider mating systems. Advances in Insect Physiology.

[29] Snell-Rood EC (2013). An overview of the evolutionary causes and consequences of behavioural plasticity. Anim. Behav..

[30] Shelly TE, Bailey WJ (1992). Experimental manipulation of mate choice by male katydids: the effect of female encounter rate. Behav. Ecol. Sociobiol..

[31] Heubel KU, Lindström K, Kokko H (2008). Females increase current reproductive effort when future access to males is uncertain. Biol. Lett..

[32] Lehmann GU (2007). Density-dependent plasticity of sequential mate choice in a bushcricket (Orthoptera: Tettigoniidae). Aust. J. Zool..

[33] Palokangas P, Alatalo RV, Korpimäki E (1992). Female choice in the kestrel under different availability of mating options. Anim. Behav..

[34] MacLeod, E. C. New insights in the evolutionary maintenance of male mate choice behaviour using the western black widow, *Latrodectus hesperus*. Doctoral dissertation, University of Toronto (2013).

[35] Salomon M, Vibert S, Bennett RG (2010). Habitat use by western black widow spiders (*Latrodectus hesperus*) in coastal British Columbia: evidence of facultative group living. Can. J. Zool..

[36] Johnson JC, Trubl P, Blackmore V, Miles L (2011). Male black widows court well-fed females more than starved females: silken cues indicate sexual cannibalism risk. Anim. Behav..

[37] Herberstein ME, Painting CJ, Holwell GI (2017). Scramble competition polygyny in terrestrial arthropods. Adv. Stud. Behav..

[38] Scott CE, McCann S, Andrade MCB (2019). Male black widows parasitize mate-searching effort of rivals to find females faster. Proc. Roy. Soc. B.

[39] Kasumovic MM, Andrade MCB (2004). Discrimination of airborne pheromones by mate-searching male western black widow spiders (*Latrodectus hesperus*): species-and population-specific responses. Can. J. Zool..

[40] Scott C, Kirk D, McCann S, Gries G (2015). Web reduction by courting male black widows renders pheromone-emitting females’ webs less attractive to rival males. Anim. Behav..

[41] Scott CE, Anderson AG, Andrade MCB (2018). A review of the mechanisms and functional roles of male silk use in spider courtship and mating. J. Arachnol..

[42] Kasumovic MM, Andrade MCB (2006). Male development tracks rapidly shifting sexual versus natural selection pressures. Curr. Biol..

[43] Kasumovic MM, Brooks RC, Andrade MCB (2009). Body condition but not dietary restriction prolongs lifespan in a semelparous capital breeder. Biol. Lett..

[44] Stoltz JA, Andrade MCB, Kasumovic MM (2012). Developmental plasticity in metabolic rates reinforces morphological plasticity in response to social cues of sexual selection. J. Insect Physiol..

[45] Kaston BJ (1970). Comparative biology of American black widow spiders. Trans. San Diego Soc. Nat. Hist..

[46] Dore AA (2018). The role of complex cues in social and reproductive plasticity. Behav. Ecol. Sociobiol..

[47] Emlen ST, Oring LW (1977). Ecology, sexual selection, and the evolution of mating systems. Science.

[48] Stoltz JA, Hanna R, Andrade MCB (2010). Longevity cost of remaining unmated under dietary restriction. Funct. Ecol..

[49] Jennions MD, Petrie M (2000). Why do females mate multiply? A review of the genetic benefits. Biol. Rev..

[50] Pitcher TE, Neff BD, Rodd FH, Rowe L (2003). Multiple mating and sequential mate choice in guppies: females trade up. Proc. Roy. Soc. B.

[51] Fowler‐Finn KD, Rodríguez RL (2012). Experience‐mediated plasticity in mate preferences: mating assurance in a variable environment. Evolution.

[52] Andrade, M. C. B. & MacLeod, E. C. Potential for CFC in black widows (genus *Latrodectus*): mechanisms and social context. In *Cryptic female choice in arthropods*: *patterns, mechanisms and prospects* (eds AV Peretti, A Aisenberg), pp. 27–53 (Springer, 2015).

[53] Prenter J, MacNeil C, Elwood RW (2006). Sexual cannibalism and mate choice. Anim. Behav..

[54] Johnson JC (2005). Cohabitation of juvenile females with mature males promotes sexual cannibalism in fishing spiders. Behav. Ecol..

[55] Baruffaldi L, Andrade MCB (2015). Contact pheromones mediate male preference in black widow spiders: avoidance of hungry sexual cannibals?. Anim. Behav..

[56] Lim H, Greenfield MD (2006). Female pheromonal chorusing in an arctiid moth, *Utetheisa ornatrix*. Behav. Ecol..

[57] Rehermann G, Altesor P, McNeil JN, González A (2016). Conspecific females promote calling behavior in the noctuid moth, *Pseudaletia adultera*. Entomol. Exp. Appl..

[58] Andrade MCB (2019). Sexual selection and social context: Web-building spiders as emerging models for adaptive plasticity. Adv. Stud. Behav..

[59] Pompilio L, Franco MG, Chisari LB, Manrique G (2016). Female choosiness and mating opportunities in the blood-sucking bug *Rhodnius prolixus*. Behaviour.

[60] Westerman EL, Drucker CB, Monteiro A (2014). Male and female mating behavior is dependent on social context in the butterfly *Bicyclus anynana*. J. Insect Behav..

[61] MacLeod EC, Andrade MCB (2014). Strong, convergent male mate choice along two preference axes in field populations of black widow spiders. Anim. Behav..

[62] Ross K, Smith RL (1979). Aspects of the courtship behavior of the black widow spider, *Latrodectus hesperus* (Araneae: Theridiidae), with evidence for the existence of a contact sex pheromone. J. Arachnol..

[63] Scott C, Vibert S, Gries G (2012). Evidence that web reduction by western black widow males functions in sexual communication. Can. Entomol..

[64] Scott, C., McCann, S., Gries, R., Khaskin, G. & Gries, G. *N*-3-methylbutanoyl-*O*-methylpropanoyl-*L*-serine methyl ester–pheromone component of western black widow females. *J. Chem. Ecol.* **41**, 465–472 (2015). 10.1007/s10886-015-0582-x 25940849

[65] Baruffaldi, L., Andrade, M. C. B. Immature mating as a tactic of polygynous male western widow spiders. *Sci. Nat.* **107**, 6 (2020). 10.1007/s00114-019-1663-4 31900596

[66] R Core Team. *R: A language and environment for statistical computing*. R Foundation for Statistical Computing, Vienna, Austria. https://www.R-project.org/ (2019).

[67] Kosmidis, I. *brglm: Bias Reduction in Binary-Response Generalized Linear Models*. R package version 0.6.2. https://cran.r-project.org/package=brglm (2019).

