# Supplementary material for: Black widows as plastic wallflowers: female choosiness increases with indicators of high mate availability in a natural population
Source: Sci Rep. 2020 Jun 2;10:8981. doi: 10.1038/s41598-020-65985-z (PMC7265538; doi:10.1038/s41598-020-65985-z)

SM2-Rcode.R

cataranea

2020-02-27

```r
# analyses for "Black widows as plastic wallflowers: female choosiness
# increases with indicators of high mate availability in a natural population"

library(tinytex)
library(brglm)
```

```
## Loading required package: profileModel

## 'brglm' will gradually be superseded by 'brglm2' (https://cran.r-project.org/package=brglm2), which p
```

```r
# read data file
sub = read.csv("SM3-data.csv", header = T)
head(sub)
```

```
##   trt cage pdays mdays males.p males.m total.males any.males
## 1   A   1A    22    17       0      14          14         1
## 2   A   2A    12    31       0       8           8         1
## 3   A   3A    20    23       0       1           1         1
## 4   A   4A    43     0       0      NA           0         0
## 5   A   5A    19    24       0       0           0         0
## 6   A   6A    10    33       0      14          14         1
##   any.males.before any.males.after days.to.male.after mating.trial
## 1                0               1                  3            Y
## 2                0               1                  3            Y
## 3                0               1                 12            Y
## 4                0               0                 NA            N
## 5                0               0                 NA            Y
## 6                0               1                  4            Y
##   cannibalism precopcann sacs viablesacsYN mated
## 1           N          N    1            1     Y
## 2           Y          Y    0            0     N
## 3           Y          Y    0            0     N
## 4        <NA>       <NA>    0            0  <NA>
## 5           N          N    0            0     Y
## 6           N          N    2            1     Y
```

```r
str(sub)
```

```
## 'data.frame':    40 obs. of  17 variables:
##  $ trt               : Factor w/ 2 levels "A","B": 1 1 1 1 1 1 1 1 1 1 ...
##  $ cage              : Factor w/ 40 levels "10A","10B","11A",..: 21 25 27 29 31 33 35 37 39 1 ...
##  $ pdays             : int  22 12 20 43 19 10 43 17 29 3 ...
##  $ mdays             : int  17 31 23 0 24 33 0 26 9 40 ...
##  $ males.p           : int  0 0 0 0 0 0 0 0 0 0 ...
##  $ males.m           : int  14 8 1 NA 0 14 NA 0 0 0 ...
```

```
##  $ total.males       : int  14 8 1 0 0 14 0 0 0 0 ...
##  $ any.males         : int  1 1 1 0 0 1 0 0 0 0 ...
##  $ any.males.before  : int  0 0 0 0 0 0 0 0 0 0 ...
##  $ any.males.after   : int  1 1 1 0 0 1 0 0 0 0 ...
##  $ days.to.male.after: int  3 3 12 NA NA 4 NA NA NA NA ...
##  $ mating.trial      : Factor w/ 2 levels "N","Y": 2 2 2 1 2 2 1 2 2 2 ...
##  $ cannibalism       : Factor w/ 2 levels "N","Y": 1 2 2 NA 1 1 NA 2 1 1 ...
##  $ precopcann        : Factor w/ 2 levels "N","Y": 1 2 2 NA 1 1 NA 1 1 1 ...
##  $ sacs              : int  1 0 0 0 0 2 0 2 0 0 ...
##  $ viablesacsYN      : int  1 0 0 0 0 1 0 1 0 0 ...
##  $ mated             : Factor w/ 2 levels "N","Y": 2 1 1 NA 2 2 NA 2 1 2 ...
```

```
# description of variables:

# trt = treatment (A = clustered, B = isolated)
# cage = cage ID for each experimental female
# pdays = number of days female spent as immature
# mdays = number of days female spent as mature
# males.p = number of males who visited a female when she was still immature
#           (in her penultimate instar)
# males.m = number of males who visited a female after her moult to maturity
# total.males = total number of males who visited a female during the field
#               experiment
# any.males = whether female was visited by any males (1) or not (0) during
#             the field experiment
# any.males.before = whether female was visited by any males (1) or not (0)
#                    before maturity
# any.males.after = whether female was visited by any males (1) or not (0)
#                   after maturity
# days.to.male.after = number of days after maturity before the first male
#                      arrived at a female's cage
# mating.trial = whether female was used in a mating trial (Y) or not (N)
# cannibalism = whether the female engaged in cannibalism (Y) or not (N) during
#               the mating trial
# precopcann = whether the female engaged in pre-copulatory cannibalism (Y)
#              or not (N) during the mating trial
# sacs = number of egg sacs produced by the female before her natural death
# viablesacsYN = whether the female produced any egg sacs from which live
#                spiderlings emerged (Y) or not (N)
# mated = whether the female mated (Y) or not (N) during the mating trial



# compare total number of males that visited females in each treatment

par(mfrow = c(2,1))
par(mar = c(4,4,1,1))
hist(sub$total.males[sub$trt == "A"], main = "clumped",
     breaks = c(0:30), right = F,
     xlim = c(0,30), ylim = c(0,10), include.lowest = F)
hist(sub$total.males[sub$trt == "B"], main = "isolated",
     breaks = c(0:30), right = F,
     xlim = c(0,30), ylim = c(0,10), include.lowest = F)
```

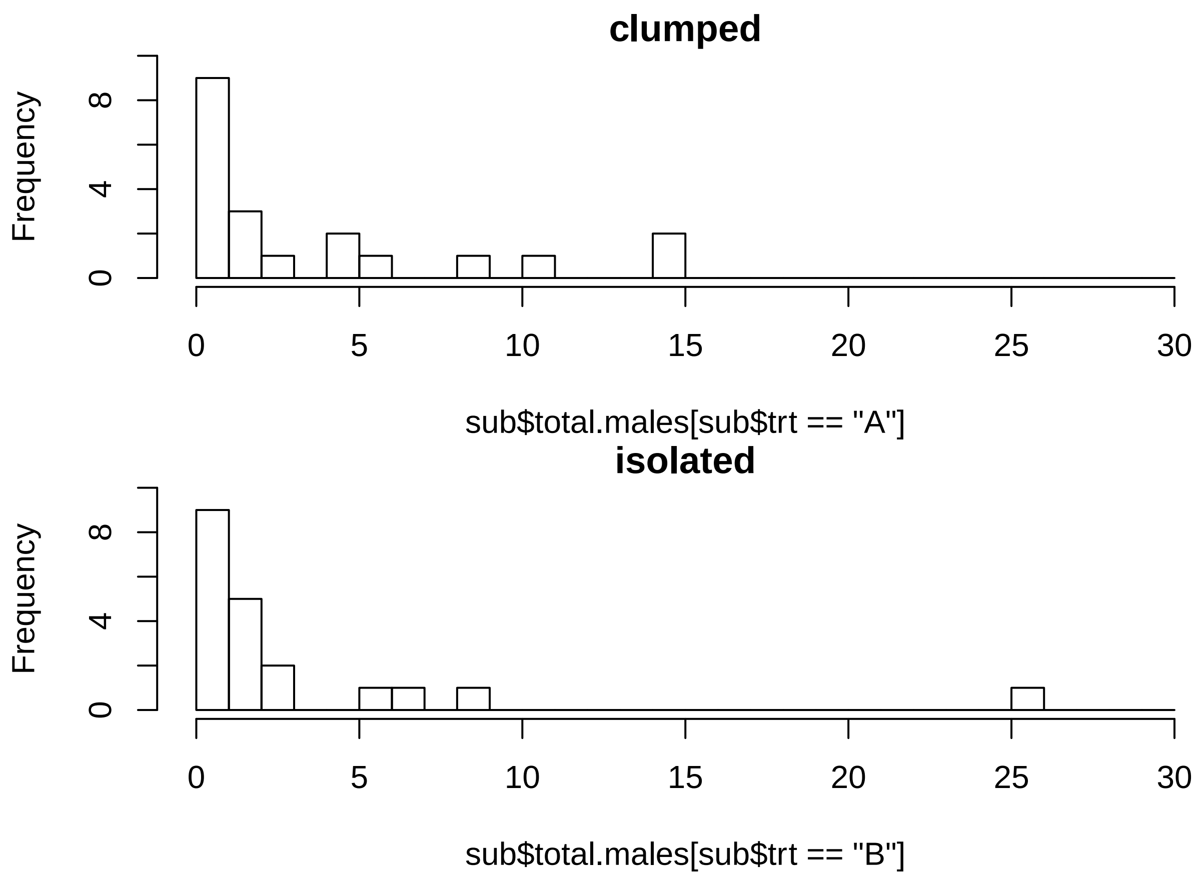


```r
mean(sub$total.males[sub$trt == "A"])
```

```
## [1] 3.2
```

```r
mean(sub$total.males[sub$trt == "B"])
```

```
## [1] 2.65
```

```r
median(sub$total.males[sub$trt == "A"])
```

```
## [1] 1
```

```r
median(sub$total.males[sub$trt == "B"])
```

```
## [1] 1
```

```r
wilcox.test(sub$total.males[sub$trt == "A"], sub$total.males[sub$trt == "B"])
```

```
## Warning in wilcox.test.default(sub$total.males[sub$trt == "A"],
## sub$total.males[sub$trt == : cannot compute exact p-value with ties
```

```
##
##  Wilcoxon rank sum test with continuity correction
##
## data:  sub$total.males[sub$trt == "A"] and sub$total.males[sub$trt == "B"]
## W = 214, p-value = 0.7004
## alternative hypothesis: true location shift is not equal to 0
```

```
# compare number of females were visited by any males before maturity
table(sub$trt, sub$any.males.before)
```

```
##
##      0  1
##   A 20  0
##   B 17  3
```

```
fisher.test(table(sub$trt, sub$any.males.before))
```

```
##
##  Fisher's Exact Test for Count Data
##
## data:  table(sub$trt, sub$any.males.before)
## p-value = 0.2308
## alternative hypothesis: true odds ratio is not equal to 1
## 95 percent confidence interval:
##  0.4266538       Inf
## sample estimates:
## odds ratio
##        Inf
```

```
# compare number of females were visited by any males after maturity
table(sub$trt, sub$any.males.after)
```

```
##
##      0  1
##   A  9 11
##   B 11  9
```

```
fisher.test(table(sub$trt, sub$any.males.after))
```

```
##
##  Fisher's Exact Test for Count Data
##
## data:  table(sub$trt, sub$any.males.after)
## p-value = 0.7524
## alternative hypothesis: true odds ratio is not equal to 1
## 95 percent confidence interval:
##  0.1612489 2.7529622
## sample estimates:
## odds ratio
##  0.6762344
```

```r
# compare timing of first male arrival between treatments
mean(sub$days.to.male.after[sub$trt == "A"], na.rm = T)
```

```
## [1] 8.090909
```

```r
mean(sub$days.to.male.after[sub$trt == "B"], na.rm = T)
```

```
## [1] 12.75
```

```r
median(sub$days.to.male.after[sub$trt == "A"], na.rm = T)
```

```
## [1] 4
```

```r
median(sub$days.to.male.after[sub$trt == "B"], na.rm = T)
```

```
## [1] 10
```

```r
min(sub$days.to.male.after[sub$trt == "A"], na.rm = T)
```

```
## [1] 1
```

```r
min(sub$days.to.male.after[sub$trt == "B"], na.rm = T)
```

```
## [1] 4
```

```r
max(sub$days.to.male.after[sub$trt == "A"], na.rm = T)
```

```
## [1] 20
```

```r
max(sub$days.to.male.after[sub$trt == "B"], na.rm = T)
```

```
## [1] 26
```

```r
hist(sub$days.to.male.after[sub$trt == "A"], main = "clustered",
     xlim = c(0, 28), ylim = c(0, 8), breaks = 10)
hist(sub$days.to.male.after[sub$trt == "B"], main = "isolated",
     xlim = c(0, 28), ylim = c(0, 8), breaks = 10)
```

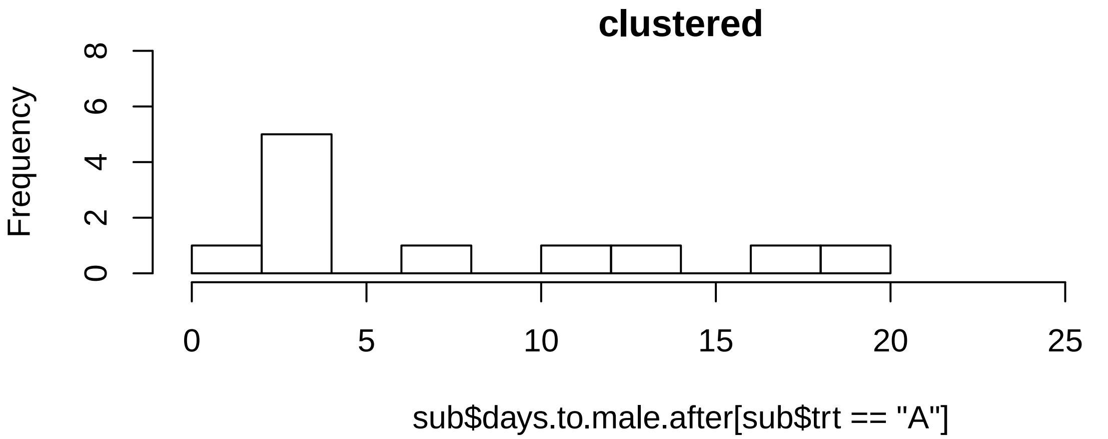

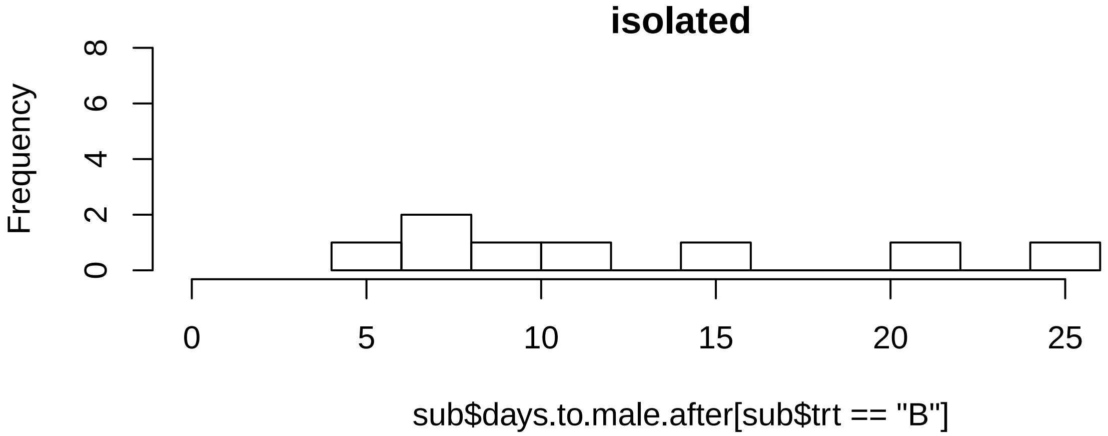


```
# (log-transform data to meet assumption of normality for t-test)
hist(log(sub$days.to.male.after[sub$trt == "A"]), main = "clustered",
     xlim = c(0, 5), ylim = c(0, 8), breaks = 8)
hist(log(sub$days.to.male.after[sub$trt == "B"]), main = "isolated",
     xlim = c(0, 5), ylim = c(0, 8), breaks = 5)
```

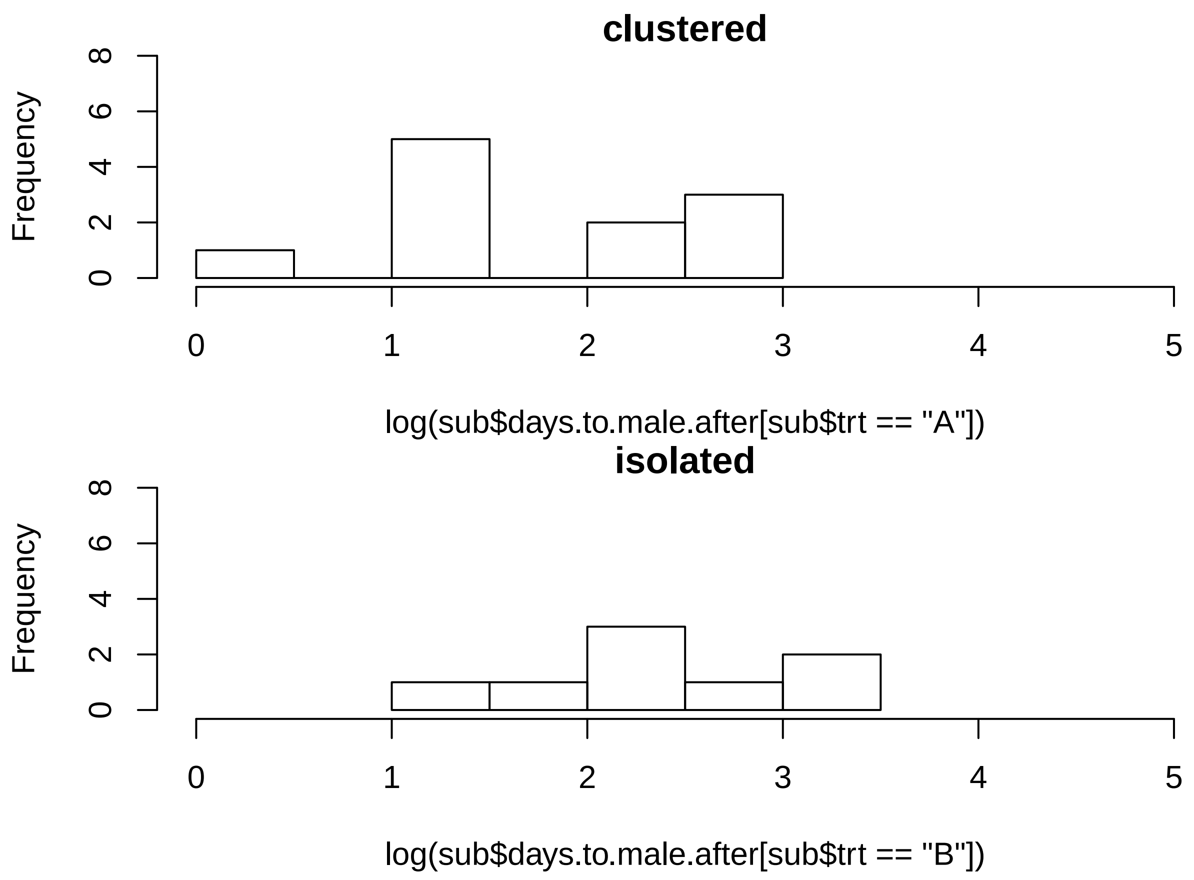


```r
t.test(log(days.to.male.after) ~ trt, data = sub, alternative = "less", var.equal = F)
```

```
##
##  Welch Two Sample t-test
##
## data:  log(days.to.male.after) by trt
## t = -1.8187, df = 16.901, p-value = 0.04336
## alternative hypothesis: true difference in means is less than 0
## 95 percent confidence interval:
##         -Inf -0.02799445
## sample estimates:
## mean in group A mean in group B
##        1.736434        2.385247
```

```r
exp(1.736)
```

```
## [1] 5.6746
```

```r
exp(2.385)
```

```
## [1] 10.85906
```

```r
# number of males arriving within 2 days of 1st male
A = c(3,2,1,5,1,1,1,2,2,1,2)
B = c(2,1,1,1,1,1,1,1)


hist(A, breaks = seq(min(A) - 0.5, max(A) + 0.5, by = 1),
     xlim = c(0.5, 6), ylim = c(0, 8), main = "clustered")
hist(B, breaks = seq(min(B) - 0.5, max(B) + 0.5, by = 1),
     xlim = c(0.5, 6), ylim = c(0, 8), main = "isolated")
```

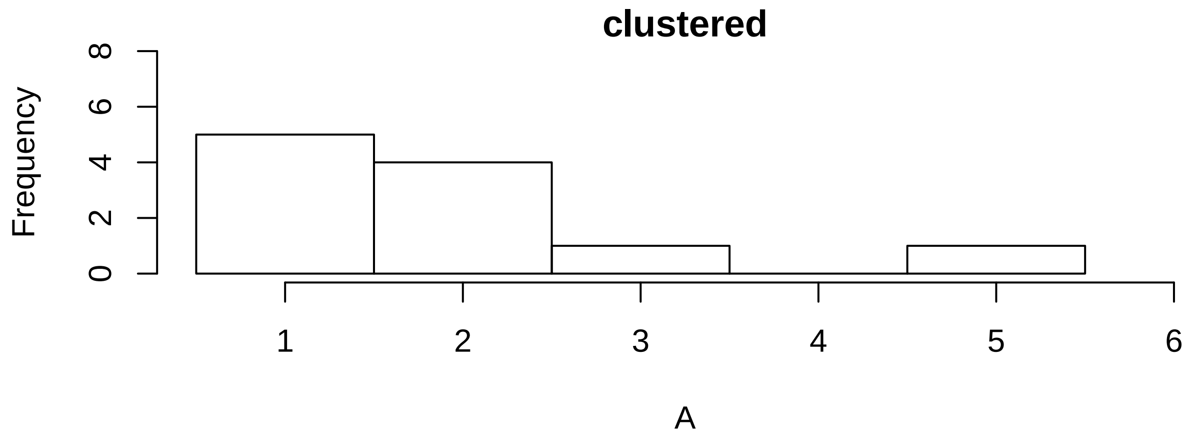

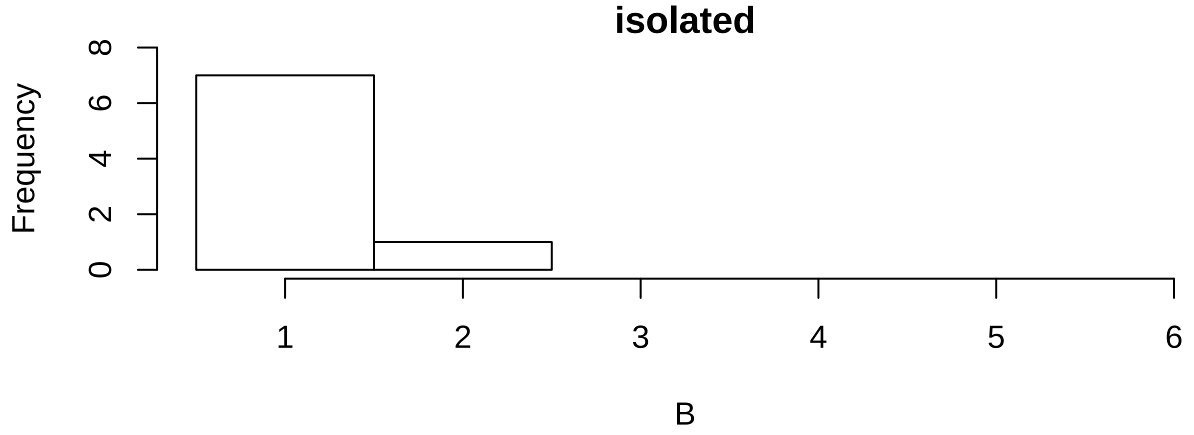


```r
mean(A)
```

```
## [1] 1.909091
```

```r
mean(B)
```

```
## [1] 1.125
```

```r
median(A)
```

```
## [1] 2
```

```r
median(B)
```

```
## [1] 1
```

```r
wilcox.test(A, B, alternative = "greater")
```

```
## Warning in wilcox.test.default(A, B, alternative = "greater"): cannot
## compute exact p-value with ties
```

```
##
##  Wilcoxon rank sum test with continuity correction
##
## data:  A and B
## W = 63.5, p-value = 0.03331
## alternative hypothesis: true location shift is greater than 0
```

```r
# Make figure 1

par(mar = c(6.5, 4.5, 1, 1), oma = c(0, 0, 0, 0))
par(mfrow = c(2, 2))
par(las = 1, lwd = 1.5)

hist(sub$days.to.male.after[sub$trt == "A"], #main = "clumped",
     xaxt = "n", yaxt = "n",
     xlim = c(0, 28), ylim = c(0, 8), breaks = 10,
     col = "grey", xaxs = "i", yaxs = "i",
     xlab = "", ylab = "", main = "")

axis(1, at = seq(0, 28, by = 2), labels = rep("", 15),
     mgp = c(3, 1.4, 0), cex.axis = 1.5, tck = -0.04, lwd = 1.5,lwd.ticks = 1.5)
axis(2, at = seq(0, 8, by = 1), labels = rep("", 9),
     mgp = c(3, 1, 0), cex.axis = 1.5, tck = -0.05, lwd = 1.5, lwd.ticks = 1.5)
axis(2, at = seq(0, 8, by = 2), labels = seq(0, 8, by = 2),
     mgp = c(3, 1, 0), cex.axis = 1.5, tck = -0.04, lwd = 1.5, lwd.ticks = 1.5)
title(ylab = "Frequency", line = 3, cex.lab = 1.5)
abline(v = median(sub$days.to.male.after[sub$trt == "A"], na.rm = T), lwd = 2, col = "red")
text(15, 5, "clumped", pos = 4, cex = 1.5)
text(25, 7, "a", pos = 4, cex = 1.5)

hist(A, breaks = seq(min(A) - 0.5, max(A) + 0.5, by = 1),
     xaxt = "n", yaxt = "n",
     xlim = c(0.5, 10.5), ylim = c(0, 8),
     col = "grey", xaxs = "i", yaxs = "i",
     xlab = "", ylab = "", main = "")

axis(1, at = seq(1, 5, by = 1), labels = rep("", 5),
     mgp = c(3, 1.4, 0), cex.axis = 1.5, tck = -0.04, lwd = 1.5, lwd.ticks = 1.5)
axis(2, at = seq(0, 8, by = 1), labels = rep("", 9),
     mgp = c(3, 1, 0), cex.axis = 1.5, tck = -0.04, lwd = 1.5, lwd.ticks = 1.5)
abline(v = median(A, na.rm = T), lwd = 2, col = "red")
box(bty = "l")
text(2, 5, "clumped", pos = 4, cex = 1.5)
```

```r
text(5, 7, "b", pos = 4, cex = 1.5)

hist(sub$days.to.male.after[sub$trt == "B"], #main = "clumped",
     xaxt = "n", yaxt = "n",
     xlim = c(0, 28), ylim = c(0, 8), breaks = 10,
     col = "grey", xaxs = "i", yaxs = "i",
     xlab = "", ylab = "", main = "")

axis(1, at = seq(0, 28, by = 2), labels = rep("", 15),
     mgp = c(3, 1.4, 0), cex.axis = 1.5, tck = -0.04, lwd = 1.5,lwd.ticks = 1.5)
axis(1, at = seq(0, 28, by = 4), labels = seq(0, 28, by = 4),
     mgp = c(3, 1, 0), cex.axis = 1.5, tck = 0, lwd = 1.5, lwd.ticks = 1.5)
axis(2, at = seq(0, 8, by = 1), labels = rep("", 9),
     mgp = c(3, 1, 0), cex.axis = 1.5, tck = -0.04, lwd = 1.5, lwd.ticks = 1.5)
axis(2, at = seq(0, 8, by = 2), labels = seq(0, 8, by = 2),
     mgp = c(3, 1, 0), cex.axis = 1.5, tck = -0.04, lwd = 1.5, lwd.ticks = 1.5)
title(ylab = "Frequency", line = 3, cex.lab = 1.5)
abline(v = median(sub$days.to.male.after[sub$trt == "B"], na.rm = T), lwd = 2, col = "red")
text(10, 5, "isolated", pos = 4, cex = 1.5)
text(25, 7.5, "c", pos = 4, cex = 1.5)

title(xlab = "Timing of first male arrival\n(days after female's moult)", line = 4, cex.lab = 1.5)

hist(B, breaks = seq(min(B) - 0.5, max(B) + 0.5, by = 1),
     xaxt = "n", yaxt = "n",
     xlim = c(0.5, 10.5), ylim = c(0, 8),
     col = "grey", xaxs = "i", yaxs = "i",
     xlab = "", ylab = "", main = "")

axis(1, at = seq(1, 5, by = 1), labels = seq(1, 5, by = 1),
     mgp = c(3, 1, 0), cex.axis = 1.5, tck = -0.04, lwd = 1.5, lwd.ticks = 1.5)
axis(2, at = seq(0, 8, by = 1), labels = rep("", 9),
     mgp = c(3, 1, 0), cex.axis = 1.5, tck = -0.04, lwd = 1.5, lwd.ticks = 1.5)
abline(v = median(B, na.rm = T), lwd = 2, col = "red")
box(bty = "l")
text(2, 5, "clumped", pos = 4, cex = 1.5)
text(5, 7, "d", pos = 4, cex = 1.5)

title(xlab = "Number of males", line = 3.5, cex.lab = 1.5)
```

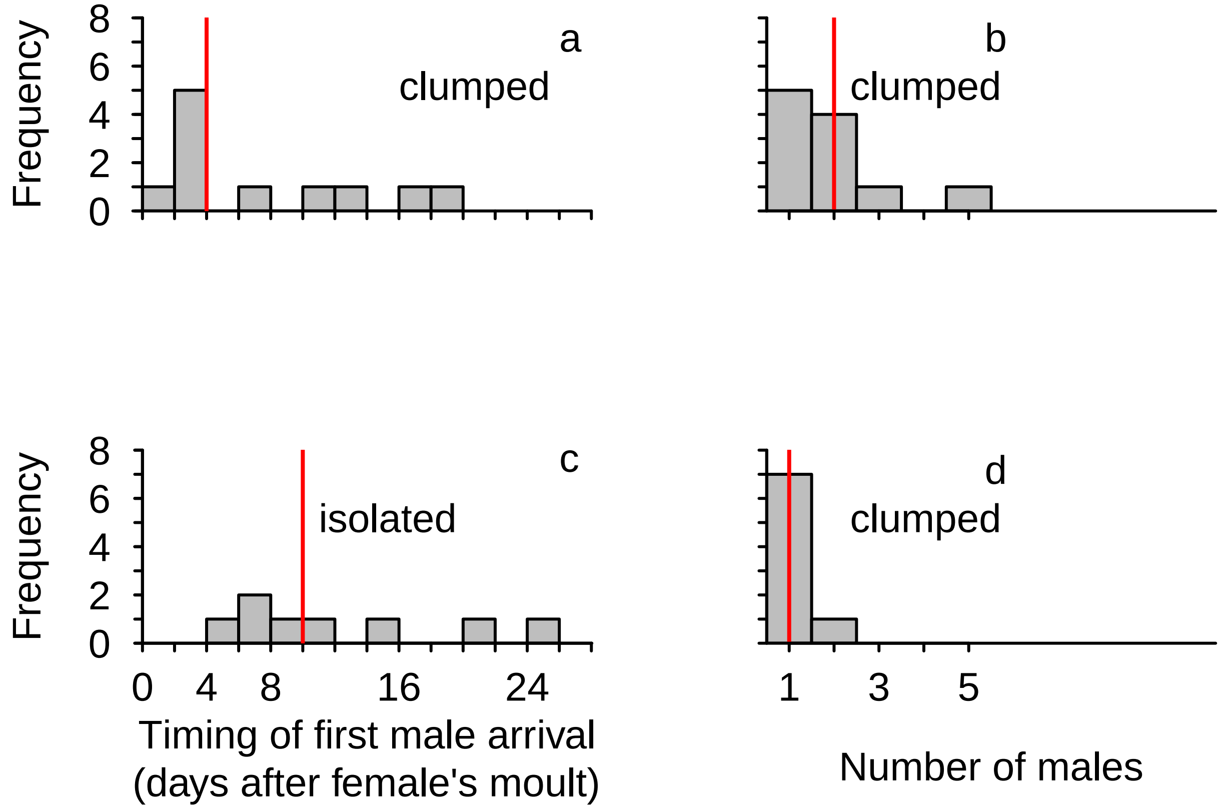


```
###############################
# Now analyze mating trial data
###############################

# first get only data for females used in mating trials
mt = sub[sub$mating.trial == "Y", ]
head(mt)
```

```
##   trt cage pdays mdays males.p males.m total.males any.males
## 1   A   1A    22    17       0      14          14         1
## 2   A   2A    12    31       0       8           8         1
## 3   A   3A    20    23       0       1           1         1
## 5   A   5A    19    24       0       0           0         0
## 6   A   6A    10    33       0      14          14         1
## 8   A   8A    17    26       0       0           0         0
##   any.males.before any.males.after days.to.male.after mating.trial
## 1                0               1                  3            Y
## 2                0               1                  3            Y
## 3                0               1                 12            Y
## 5                0               0                 NA            Y
## 6                0               1                  4            Y
## 8                0               0                 NA            Y
##   cannibalism precopcann sacs viablesacsYN mated
## 1           N          N    1            1     Y
## 2           Y          Y    0            0     N
## 3           Y          Y    0            0     N
```

```
## 5           N          N    0            0     Y
## 6           N          N    2            1     Y
## 8           Y          N    2            1     Y
```

```r
# compare mating success between treatments
table(mt$trt, mt$mated)
```

```
##
##      N  Y
##   A  5 10
##   B  0 15
```

```r
fisher.test(table(mt$trt, mt$mated), alternative = "greater")
```

```
##
##  Fisher's Exact Test for Count Data
##
## data:  table(mt$trt, mt$mated)
## p-value = 0.02107
## alternative hypothesis: true odds ratio is greater than 1
## 95 percent confidence interval:
##  1.456402      Inf
## sample estimates:
## odds ratio
##        Inf
```

```r
# compare total cannibalism between treatments
table(mt$trt, mt$cannibalism)
```

```
##
##      N  Y
##   A 10  5
##   B 13  2
```

```r
fisher.test(table(mt$trt, mt$cannibalism), alternative = "less")
```

```
##
##  Fisher's Exact Test for Count Data
##
## data:  table(mt$trt, mt$cannibalism)
## p-value = 0.1949
## alternative hypothesis: true odds ratio is less than 1
## 95 percent confidence interval:
##  0.000000 1.905785
## sample estimates:
## odds ratio
##  0.3200303
```

```r
# compare age of females (days since moult to maturity) by treatment
par(mfrow = c(2,1))
hist(sub$mdays[sub$trt == "A"])
hist(sub$mdays[sub$trt == "B"])
```

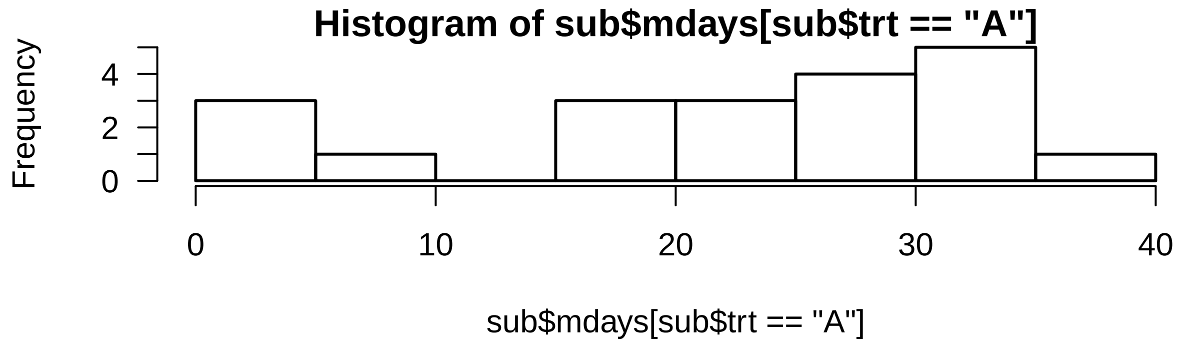

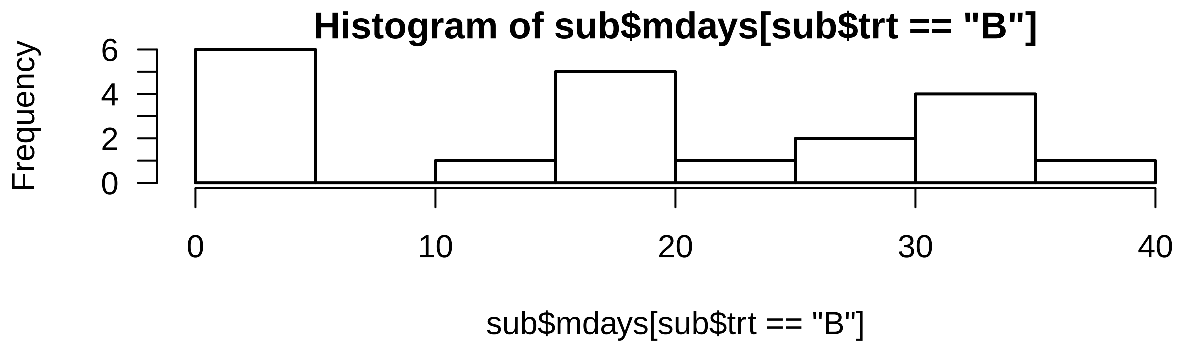


```r
wilcox.test(sub$mdays[sub$trt == "A"], sub$mdays[sub$trt == "B"])
```

```
## Warning in wilcox.test.default(sub$mdays[sub$trt == "A"], sub$mdays[sub$trt
## == : cannot compute exact p-value with ties
```

```
##
##  Wilcoxon rank sum test with continuity correction
##
## data:  sub$mdays[sub$trt == "A"] and sub$mdays[sub$trt == "B"]
## W = 241, p-value = 0.2701
## alternative hypothesis: true location shift is not equal to 0
```

```r
# logistic regression analyses (using Firth's bias adjustment method)

# effect of treatment, number of males, and female age on whether or not a
# female copulated at least once
mating = brglm(mated ~ trt + mdays + total.males, data = mt)
summary(mating)
```

```
##
## Call:
## brglm(formula = mated ~ trt + mdays + total.males, data = mt)
##
##
```

```
## Coefficients:
##              Estimate Std. Error z value Pr(>|z|)
## (Intercept) -1.65388    1.78919  -0.924   0.3553
## trtB         3.48788    1.73366   2.012   0.0442 *
## mdays        0.07544    0.06868   1.098   0.2720
## total.males  0.08935    0.12669   0.705   0.4806
## ---
## Signif. codes:  0 '***' 0.001 '**' 0.01 '*' 0.05 '.' 0.1 ' ' 1
##
## (Dispersion parameter for binomial family taken to be 1)
##
##     Null deviance: 20.215  on 29  degrees of freedom
## Residual deviance: 17.244  on 26  degrees of freedom
## Penalized deviance: 7.29615
## AIC:  25.244
```

```r
# effect of treatment, number of males, and female age on whether or not a
# female engaged in pre-copulatory cannibalism
pccann = brglm(precopcann ~ trt + mdays + total.males, data = mt)
summary(pccann)
```

```
##
## Call:
## brglm(formula = precopcann ~ trt + mdays + total.males, data = mt)
##
##
## Coefficients:
##              Estimate Std. Error z value Pr(>|z|)
## (Intercept) -0.42606    1.72050  -0.248    0.804
## trtB        -2.04826    1.44119  -1.421    0.155
## mdays       -0.02812    0.06687  -0.420    0.674
## total.males -0.00177    0.11223  -0.016    0.987
##
## (Dispersion parameter for binomial family taken to be 1)
##
##     Null deviance: 13.732  on 29  degrees of freedom
## Residual deviance: 16.323  on 26  degrees of freedom
## Penalized deviance: 5.8982
## AIC:  24.323
```

```r
# Check that rate of reproductive failure (proportion of females that copulated at
# least once but did not produce any offspring) did not differ between treatments

rf = matrix(c(2, 13,
              4, 6),
            nrow = 2, ncol = 2, byrow = T)
rownames(rf) = c("isolated", "clustered")
colnames(rf) = c("no spiderlings", "spiderlings")
rf
```

```
##           no spiderlings spiderlings
## isolated               2          13
## clustered              4           6
```

```r
fisher.test(rf)
```

```
##
##  Fisher's Exact Test for Count Data
##
## data:  rf
## p-value = 0.1753
## alternative hypothesis: true odds ratio is not equal to 1
## 95 percent confidence interval:
##  0.01755607 2.27175524
## sample estimates:
## odds ratio
##  0.2461042
```

```r
# summary table of cannibalism for plotting
canntab = matrix(c(3, 2,
                   0, 2),
                 byrow = F, 2, 2)
canntab
```

```
##      [,1] [,2]
## [1,]    3    0
## [2,]    2    2
```

```r
rownames(canntab) = c("pre-cop", "post-cop")
colnames(canntab) = c("A", "B")
canntab
```

```
##          A B
## pre-cop  3 0
## post-cop 2 2
```

```r
# Make Figure 2
par(mfrow = c(2, 1))
par(las = 1) # make all labels vertical
par(lwd = 1.5)

par(mar = c(1, 6, 5, 1))
barplot(c(10, 15), beside = F, horiz = F, width = 0.5, space = 0.5,
        col = c("grey30"), # colour bars
        xaxt = "n", # plot the data with no y-axis labels
        border = T, cex.names = 1.25, cex.axis= 1.25,
        xlim = c(0, 1.75), ylim = c(0, 15),
        xaxt = "n", yaxt = "n", yaxs = "i", xaxs = "i"
)


box(bty = "l", lwd = 1.5) # make an L-shaped frame with line width 1.5


# add axis labels
title(ylab = "Number of females\nthat copulated",
```

```r
      line = 2.5, # change to move closer or farther from x axis
      cex.lab= 1.25) # change to adjust text size

axis(2, mgp = c(3, 1, 0), cex.axis= 1.25,
     tck = -0.04, # this controls how far the tick sticks out
     lwd = 1.5, lwd.ticks = 1.5,
     labels = rep("", 16), at = seq(0, 15, 1))
axis(2, mgp = c(3, 1, 0), cex.axis= 1.25,
     tck = -0.04, # this controls how far the tick sticks out
     lwd = 1.5, lwd.ticks = 1.5,
     labels = seq(0, 15, 5), at = seq(0, 15, 5))
text(0, 14, "a", pos = 4, cex = 1.5)

par(mar = c(5, 6, 1, 1))
barplot(canntab, beside = F, horiz = F, width = 0.5, space = 0.5,
        col = c("black", "grey80"), # colour bars
        xaxt = "n", # plot the data with no y-axis labels
        yaxt = "n", yaxs = "i", xaxs = "i",
        border = T, cex.names = 1.25, cex.axis= 1.25,
        xlim = c(0,1.75), ylim = c(0, 15)
)

box(bty = "l", lwd = 1.5) # make an L-shaped frame with line width 1.5

# add axis labels
title(ylab = "Number of females\nthat cannibalised",
      line = 2.5, # change to move closer or farther from x axis
      cex.lab= 1.25) # change to adjust text size
axis(2, mgp = c(3, 0.75, 0), cex.axis= 1.25,
     tck = -0.04, # this controls how far the tick sticks out
     lwd = 1.5, lwd.ticks = 1.5,
     labels = rep("", 16), at = seq(0, 15, 1))
axis(2, mgp = c(3, 0.75, 0), cex.axis= 1.25,
     tck = -0.04, # this controls how far the tick sticks out
     lwd = 1.5, lwd.ticks = 1.5,
     labels = seq(0, 15, 5), at = seq(0, 15, 5))
text(0, 14, "b", pos = 4, cex = 1.5)

axis(1, mgp = c(3, 0.75, 0), cex.axis= 1.25,
     tck = 0, # this controls how far the tick sticks out
     lwd = 1.5, lwd.ticks = 1.5,
     labels = c("clustered", "isolated"), at = c(0.5, 1.25))

legend(x = 0.25, y = 14.5, # choose x,y coordinates for legend
       title = "timing of cannibalism:",
       c("post-copulatory", "pre-copulatory"), # specify legend text
       fill = c("grey80", "black"), # specify box colours
       border = T, # border around boxes
       bty = "n", # no box around legend
       cex = 1.25 , # control size of text
       x.intersp = 0.5 # change how far text is from boxes
)
```

```r
title(xlab = "Treatment", line = 2.5, cex.lab = 1.25)
```

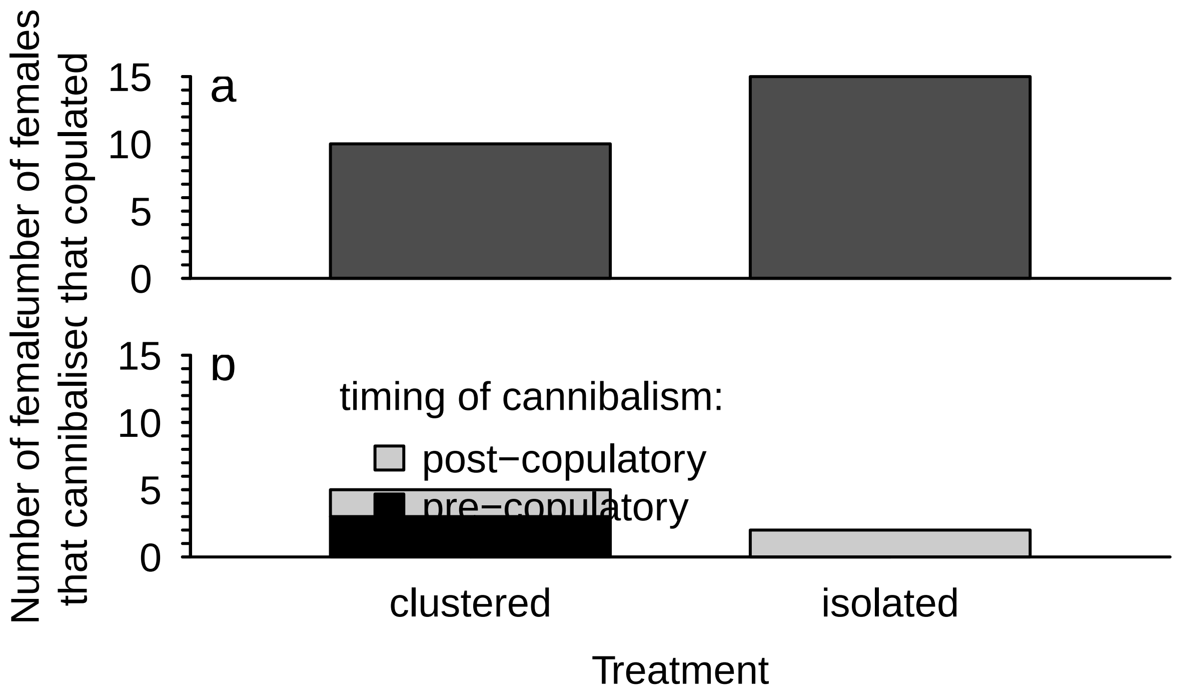

Supplement: Supplementary file 3 — Supplementary information 3. [file 41598_2020_65985_MOESM3_ESM.pdf]
