# Supplementary material for: Black widows as plastic wallflowers: female choosiness increases with indicators of high mate availability in a natural population
Source: Sci Rep. 2020 Jun 2;10:8981. doi: 10.1038/s41598-020-65985-z (PMC7265538; doi:10.1038/s41598-020-65985-z)
Supplement: Supplementary file 4 — Supplementary Material. [file 41598_2020_65985_MOESM4_ESM.docx]

**Black widows as plastic wallflowers: female choosiness increases with indicators of high mate availability in a natural population**

*Catherine E. Scott^1,2^, Sean McCann^1,2^, and Maydianne C.B. Andrade^1^

^1^ Department of Biological Sciences, University of Toronto Scarborough, M1C1A4, Canada.

^2^ Current address: Department of Biology, Acadia University, 33 Westwood Ave. Wolfville, NS, B4P 2R6

*Corresponding author: catherine.elizabeth.scott@gmail.com

**Supplementary material:**

SM1: Raw data file (.csv)

SM2: R code for all analyses (.R)

SM3: pdf version of the R code and output
